# The influence of the merger process between two tertiary hospitals in Romania on job satisfaction among staff

**DOI:** 10.3389/fpsyg.2024.1304359

**Published:** 2024-01-30

**Authors:** Codrin Dan Nicolae Ilea, Lucia Georgeta Daina, Felicia Manole, Mădalina Diana Daina, Dorel Petru Tirt, Anca Popa

**Affiliations:** ^1^Faculty of Medicine and Pharmacy, Doctoral School, University of Oradea, Oradea, Romania; ^2^Department of Psycho-Neurosciences and Recovery, Faculty of Medicine and Pharmacy, University of Oradea, Oradea, Romania; ^3^Department of Surgical Disciplines, Faculty of Medicine and Pharmacy, University of Oradea, Oradea, Romania; ^4^Faculty of Medicine and Pharmacy, University of Oradea, Oradea, Romania; ^5^Department of Endocrinology, Emergency Clinical County Hospital of Oradea, Oradea, Romania; ^6^Department of Animal Science and Agrotourism, Faculty of Environmental Protection, University of Oradea, Oradea, Romania

**Keywords:** merger, medical staff, job satisfaction, tertiary hospital, management

## Abstract

**Aim:**

The purpose of this study is to evaluate the influence of the merger process of two tertiary hospitals located in the northwest of Romania on the professional satisfaction among medical and non-medical staff and to develop a standardized satisfaction questionnaire for romanian hospitals.

**Methods:**

1750 questionnaires distributed within County Clinical Emergency Hospital Bihor (CCEHBh) ten months and one year and four months after the merger process were analyzed.

**Results:**

The percentage of staff who declare themselves satisfied with their work one year and four months after the merger is 80.14%. It has a downward trend compared to the result measured 10 months after the merger (86.14%) (χ^2^ test, *p* < 0.01). The aspects that were rated with the lowest percentage as satisfactory were the possibility of promotion (41.89%) and job security (53.38%). A statistically significant decrease was also recorded in the assessment of career prospects (from 81.49% to 74.73%, χ^2^ test, *p* < 0.0001).

**Conclusions:**

Even if there was a decrease in job satisfaction between the two evaluated periods, we can state that the general level at the last measurement is a good one (4.07 out of a maximum of 5). There was no significant difference in job satisfaction 1 year and 4 months after the merger for staff in the merged unit (4.06) compared to staff in the absorbing unit (4.09). The questionnaire applied in 2023 is one that has proven validity and reliability, being a good starting point for creating a standardized questionnaire that could be implemented in the vast majority of hospitals in Romania. The application of the questionnaire at an interval of 3–6 months would highlight the result of the implemented measures and the trend of employee satisfaction within CCEHBh.

## 1 Introduction

In the health system, restructuring is an important topic for decision-makers in this field (Spânulescu and Mocuta, 2015). However, the analysis of the effects of the reorganization process on employee satisfaction in the health field suffers from some gaps (Spânulescu and Mocuta, 2015).

Achieving the health-related outcome expected by the patient reflects the effectiveness of health services (Munteanu et al., 2021). In addition to providing quality patient services, hospitals must also be as efficient as possible from a financial point of view. Financial efficiency can be achieved through numerous methods including reorganization. One of the reorganization options encountered in the healthcare system is the merger of two or more healthcare units.

Mergers are corporate phenomena that can create severe personal trauma that can lead to psychological, physical or behavioral problems and adjustment problems for both the individual and the companies involved (Ivancevich et al., 1987). The merger process has a complex aspect with poorly defined boundaries, which involves some difficulties arising from the differences in perception of the organizational culture and perceptions about takeover, which limit the exchange of valuable information between the newly merged organizations (Fulop et al., 2005). Through the disappearance, following a merger, of some formal organizational structures, states of stress and conflict may arise, felt by some employees as a sense of loss, pain and sometimes anger related to the loss of old identities and values (Ivancevich et al., 1987; Sutton, 1987; Sanda and Adjei-Benin, 2011). Hospital mergers have a significant effect on employee sick leave frequency both in the short term and 2–4 years after the merger (Bourbonnais et al., 2005; Kjekshus et al., 2014; Ingelsrud, 2017; Cerezo-Espinosa de los Monteros et al., 2021). There is evidence of increased incidence of psychosocial stress, risk of depression, anxiety, sleep disturbances and a higher rate of retirement among staff early in the merger process (Breinegaard et al., 2017; Cerezo-Espinosa de los Monteros et al., 2021). After the merger, lower job satisfaction is associated with high turnover among medical staff (Cerezo-Espinosa de los Monteros et al., 2021). The negative effect is no longer statistically significant after one year (Kjekshus et al., 2014; Cerezo-Espinosa de los Monteros et al., 2021). Staff job satisfaction can be ensured by the fulfillment of expectations related to the benefits of the merger (Bourbonnais et al., 2005; Kjekshus et al., 2014; Lim, 2014; Cerezo-Espinosa de los Monteros et al., 2021).

A review published in 2002 of mergers in the London health system, between April 1998 and April 1999, concluded that the merger led to a period of organizational restructuring that delayed the evolution of the organization and services by at least 18 months and that the time required for restructuring was underestimated by those who initiated the mergers and those who implemented them (Fulop et al., 2002). Another major impasse in a merger is the difference in culture between the joining organizations (Fulop et al., 2002).

A study published in 2014 analyzing the influence of structural changes in the Italian National Health Service concludes that overall job satisfaction is influenced by these changes (Mascia et al., 2014). Increased job satisfaction immediately after the merger is associated with pre-merger employee engagement (Cerezo-Espinosa de los Monteros et al., 2021).

The expected benefits from a financial point of view should be weighed by the decision-makers against the effects of the merger process on the quality of the medical act and on the medical staff. A study published in the USA in 2017 analyzing the evolution of costs in hospitals that merged between 1998 and 2012 concludes that there is an average cost reduction for the hospital that was absorbed by 4-7% for the post-merger period (Schmitt, 2017).

For a successful transition following a merger, the following objectives need to be achieved: communication with employees, determination of employee concerns, avoidance of ambiguities and uncertainties, and job security (Davy et al., 1988).

The following impediments to a merger are identified in the specialized literature: inadequate integration of the organization's culture, poor management of the change process, technological compatibility and business plans, regulatory and compliance requirements, as well as the human factor (Sanda and Adjei-Benin, 2011). In the early 2000s, several articles in the literature conclude that minimal importance is given to the role of the human factor in the success of a merger (Kay and Shelton, 2000; Gunther, 2001; Schuler and Jackson, 2001; Jackson et al., 2003; Sanda and Adjei-Benin, 2011).

If the failure of some mergers can be explained in terms of financial or market factors, there are many such failed mergers that can be attributed to the human factor (Schuler and Jackson, 2001; Sanda and Adjei-Benin, 2011). Further studies demonstrate the need for units to address the issue of the human factor in the fusion process (Jackson et al., 2003; Antila, 2006; Björkman and Søderberg, 2006; Kavanagh and Ashkanasy, 2006; Guerrero, 2008; Sanda and Adjei-Benin, 2011). Merger managers are highly focused on the operational, legal and financial components of the process and less focused on the human aspect (Jackson et al., 2003; Antila, 2006; Björkman and Søderberg, 2006; Kavanagh and Ashkanasy, 2006; Guerrero, 2008; Sanda and Adjei-Benin, 2011). This approach can be considered a major weakness in understanding organizational behavior and the direct effect of the human factor on the smooth running of the merger process (Jackson et al., 2003; Antila, 2006; Björkman and Søderberg, 2006; Kavanagh and Ashkanasy, 2006; Guerrero, 2008; Sanda and Adjei-Benin, 2011). If employees question the fairness of their new organizational structure, they may develop feelings of distrust in the actions of those who manage the new entity (Daileyl and Kirk, 1992; McFarlin and Sweeney, 1992; Sanda and Adjei-Benin, 2011). This mistrust, once it arises, can be difficult to counter (Daileyl and Kirk, 1992; McFarlin and Sweeney, 1992; Sanda and Adjei-Benin, 2011).

On February 26, 2022, the merger of the two largest hospitals in the Municipality of Oradea was completed. Thus the Municipal Clinical Hospital “Dr. Gavril Curteanu” Oradea (MCHO) was annexed to the County Clinical Emergency Hospital Oradea (CCEHO). The takeover was achieved through the full incorporation of all material assets and all personnel. The expected benefits, according to city officials, were: higher funding per case treated, reduced spending on services and product purchases, and a faster patient cycle (Criş, 2022). Since it was not known exactly what the new organizational structure would look like following the merger, there were feelings of anxiety among the staff (Criş, 2022).

As of January 1, 2023, the County Clinical Emergency Hospital Oradea is transforming into the County Clinical Emergency Hospital Bihor (CCEHBh) with a new organizational structure. This aspect completes the merger process between the two hospitals initiated about ten months ago.

The present study, using the satisfaction questionnaire distributed to employees in December 2022 and then in June 2023, attempts to assess the effects of the merger process on job satisfaction in the newly formed hospital unit. The objectives pursued were the comparative evaluation of job satisfaction after the merger process and the testing of a new satisfaction questionnaire.

## 2 Materials and methods

### 2.1 Study design

In order to achieve the established objectives, the answers received to the satisfaction questionnaire periodically distributed CCEHBh employees were analyzed.

In the questionnaire applied in 2022, the assessment of job satisfaction was carried out using 14 items grouped into seven categories: collaboration with colleagues (1 item), collaboration with the direct superior (3 items), collaboration with the hospital management (3 items), motivation (1 item), the possibility of promotion (1 item), job security (3 items) and professional development (2 items). Of the 14 items, 10 were formulated as dichotomous questions, 3 items had 3 answer options (unsatisfactory, satisfactory and good) and 1 item had 4 answer options (I did not participate, unsatisfactory, satisfactory and good). People who answered “yes” to the dichotomous questions and “satisfactory” and “good” to the 3 and 4 choise questions were considered satisfied. For categories that were composed of several items, the average of the responses was used.

In an attempt to improve the evaluation of employee satisfaction, a new questionnaire was designed in 2023 using a 5-variant Likert scale as the unit. A comparison between the two questionnaires, the wording, the form of the questions and the answer options are presented in Table 1.

**Table 1:** The comparative structure of the questionnaires used.

	**2022**		**2023**	
**Category**	**Item/question**	**Answer options**	**Item/question**	**Answer options**
Collaboration with colleagues	Do you think that there is a good relationship between you as an employee and your colleagues?	Yes/No	Collaborating with colleagues is effective, based on real and open communication.	5-point Likert scale
			In exercising my profession, I cooperate constructively with my colleagues to fulfill my professional obligations.	5-point Likert scale
Collaboration with the direct superior	Is there a relationship of communication and collaboration between you and the hierarchical bosses?	Yes/No	Decisions that affect my work are communicated to me clearly and at the right time by my direct boss.	5-point Likert scale
	As an employee, do you know what the results are expected by your bosses regarding the activity you carry out?	Yes/No	I know what results my boss expects from the work I do.	5-point Likert scale
	Do you think your superior listens to you and analyzes your ideas/proposals for improvement?	Yes/No	CI believe that the boss listens to my suggestions for improvement.	5-point Likert scale
Collaboration with the hospital management	Do you think the intranet portal is useful for your activity?	Yes/No	I believe that the “Nextcloud” intranet portal is useful for my activity.	5-point Likert scale
	Do you feel you have real-time access to the data and information you need?	Yes/No	The information sent by the management of the institution is communicated to me in a timely, clear and objective manner.	5-point Likert scale
	Do you think there is a communication relationship between the hospital management and you?	Yes/No	I have real-time access to the data and information necessary to fulfill my responsibilities.	5-point Likert scale
The work itself	Are you motivated?	Yes/No	The activity I do every day is motivating.	5-point Likert scale
The possibility of promotion	Do you think that the hospital has a promotion policy for its employees?	Yes/No	I believe that the hospital has a promotion policy for its employees.	5-point Likert scale
Financial motivation			I consider the salary scale in the state medical system motivating.	5-point Likert scale
Work security	Do you feel safe in terms of the material endowment with equipment, sanitary materials, equipment, necessary to carry out your activity?	Yes/No	The provision of equipment, sanitary materials, equipment necessary to carry out my activity is adequate.	5-point Likert scale
	Do you consider workplace cleanliness to be:	Unsatisfac- tory/ Satisfactory/ Good	I consider cleanliness at the workplace appropriate.	5-point Likert scale
	Do you consider the arrangement of the workplace:	Unsatisfac- tory/ Satisfactory/ Good	The layout of the workplace leaves much to be desired	5-point Likert scale
Professional development	What do you think about your professional development within the Hospital?	Unsatisfac- tory/ Satisfactory/ Advantageous	I consider that my professional development within the Hospital is satisfactory.	5-point Likert scale
	How satisfied are you with the quality and usefulness of the training and professional development courses you attended?	I did not participate/ Unsatisfac- tory/ Satisfactory/ Good	The quality of the training and professional development courses supported by the Hospital is satisfactory.	5-point Likert scale

**Figure 1:**
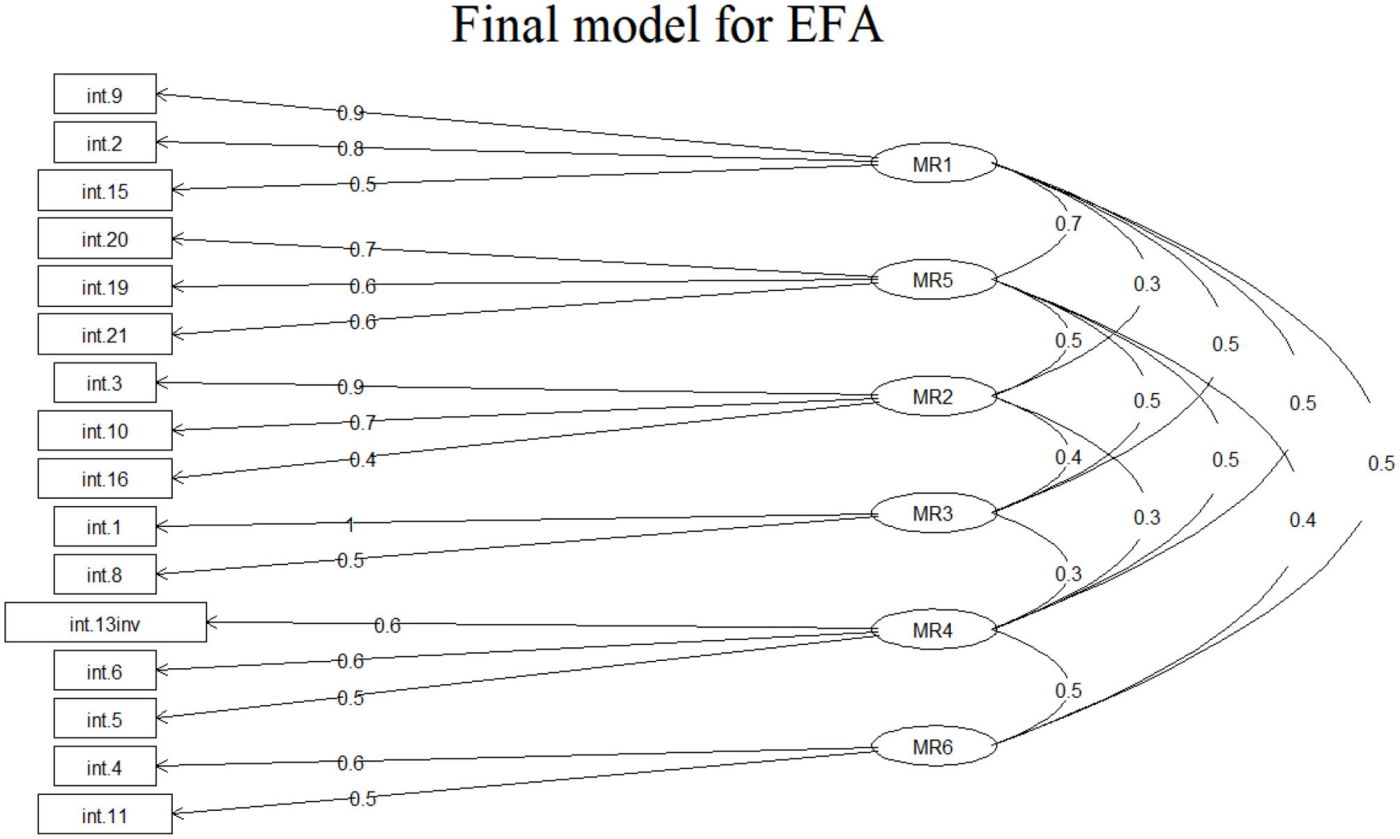
Exploratory factor analysis (EFA), item loading for the six factors.

The design of the new employee satisfaction questionnaire began by generating the preliminary items based on the information from the literature and current legislation. It should be noted that in the satisfaction questionnaire applied in public hospitals in Romania, certain aspects monitored by the National Agency for Quality Management in Health must be found. After the validation of the preliminary items by a team of five experts, the questionnaire was administered to check its validity. The questionnaire in its original form contains 22 questions grouped around six factors: collaboration with the direct superior, collaboration with colleagues, collaboration with the unit management, motivation, job security and career (professional development). The exploratory factor analysis, performed on a number of 150 questionnaires, eliminates 6 of the 22 items initially included in the questionnaire. The 16-item form yields the following results: Bartletts test for specificity *p* < 0.00001 and Kaiser-Meyer-Olkin measure = 0.9. The cumulative variance explained for the six factors was 0.66 and the loading level for each item is shown in Figure 1.

Confirmatory factor analysis demonstrates an acceptable structure of the questionnaire by obtaining the following values: comparative fit index (CFI) = 0.946, Tucker-Lewis index = 0.928, root mean square error of approximation (RMSEA) = 0.058 and standardized root mean square residual (SRMR) = 0.043. The internal reliability of the questionnaire was assessed using the Cronbach's Alpha coefficient and a result of 0.87 was obtained.

In order to be able to compare the values obtained in the two questionnaires, the people for whom the average of the answers to the items in the respective category was greater than or equal to 4 were considered satisfied for each category.

Ethical approval was obtained from the Ethics Committee of the Bihor County Emergency Clinical Hospital, Romania no. 25319/12.10.2018, and the present study was carried out in accordance with the Declaration of Helsinki.

### 2.2 Statistical analysis

To verify the statistical significance of the obtained results, the Chi-square test (χ^2^), the Fisher test, the T test, the Anova test and the Tukey's HSD test were used. The confidence interval was set at 95% with a statistical significance threshold of 0.05. Generalized nonlinear regression models were also used to test the influence of socio-economic factors on selected variables. The regression model was created using the it backward selection method. Statistical analysis was performed using the **R** program version 4.3.1.

### 2.3 Participants

CCEHBh has approximately 3,200 employees, including approximately 500 doctors and 1,400 nurses. The percentage of employed women is approximately 84% and staff with higher education represent approximately 27%.

The satisfaction questionnaire is distributed to at least 1 in 2 employees in all departments and compartments of the Hospital, so that at least one employee from each staff category in each department was asked to complete the questionnaire. Thus, in December 2022, 1,774 were distributed and 1,346 were collected, and in June 2023, 1,838 were distributed, and 1,309 were collected.

CCEHBh staff was analyzed by dividing them into four groups: doctors, nurses, auxiliary staff and technical-administrative personnel (TESA). All employees who are not doctors, nurses or administrative staff were included in the auxiliary staff category.

After eliminating incorrectly completed questionnaires, without gender or completed training level, 1,750 questionnaires remained for analysis. The distribution of the questionnaires analyzed by year according to the gender of the employee and the level of training was as follows (Table 2).

**Table 2:** Distribution by year, gender, and schooling level of the analyzed questionnaires.

**Year**	**2022**	**2023**	**Total**
No. questionnaires	1,010	740	1,750
Women %	86.04%	83.51%	84.97%
Higher education %	48.02%	55.27%	51.09%

Although staff with higher education only represent approximately 27% of the total number of employees, approximately 50% of the total questionnaires are completed by them, which could create a certain bias in the analysis (Table 2).

The annual distribution of the analyzed questionnaires for each staff group is presented in Table 3.

**Table 3:** Distribution by year and staff group of analyzed questionnaires and average age of staff.

	**Year 2022**		**Year 2023**	
**Professional Category**	**No. Question**.	**Average Age**	**No. Question**.	**Average Age**
Doctors	166	45.09 ± 10.40	153	42.62 ± 10.41
Nurses	458	44.46 ± 8.41	327	44.79 ± 8.57
Aux. staff	359	46.40 ± 8.75	223	45.14 ± 9.45
TESA	27	45.05 ± 8.34	37	48.83 ± 8.03
**Hospital**	1,010	45.28 ± 8.90	740	44.62 ± 9.27

The age of the employees who responded to the questionnaire was between 22 and 69 years old and the average age for each category of employee is presented in Table 3.

## 3 Results

The percentage of staff who declare themselves satisfied for each category and respectively with overall job satisfaction is presented in Table 4. A person who declared himself satisfied with at least 4 of the 7 subcategories was considered satisfied with overall job satisfaction.

**Table 4:** Percentage of satisfied staff.

**Category**	**Year 2022**	**vs**	**Year 2023**
	**% Satisfied**	***p***	**% Satisfied**
Collaboration with colleagues	89.70%	<*0.0001*	99.46%
Collaboration with the direct superior	85.94%	*= 0.4*	87.43%
Collaboration with the hospital management	76.93%	*= 0.5*	75.27%
The work itself	70.59%	*= 0.08*	74.46%
The possibility of promotion	40.40%	*= 0.6*	41.89%
Work security	90.99%	<*0.0001*	53.38%
Professional development	81.49%	<*0.0001*	74.73%
**Job satisfaction**	86.14%	<*0.01*	80.14%

There is a statistically significant increase in the percentage of staff who declare themselves satisfied with intercollegiate collaboration and a statistically significant decrease in the percentage of staff who declare themselves satisfied with job security and career prospects (Table 4). Approximately 60% of the staff declare themselves dissatisfied with the possibility of promotion but without a significant change between the two measurements carried out.

Job satisfaction was analyzed from the perspective of each staff group. The results presented in Table 5 show a decrease in the percentage of people who declare themselves satisfied in 2023 compared to 2022 for each category, but with a significant decrease only in the case of doctors.

**Table 5:** The percentage of staff who are satisfied with their job.

**Staff**	**Year 2022**		**vs**.	**Year 2023**	
**group**	**No**.	**% Satisfied**	***p***	**No**.	**% Satisfied**
Doctors	166	83.73%	<*0.05*	153	72.55%
Nurses	458	86.03%	*=0.1*	327	81.96%
Aux. staff	359	88.02%	*=0.1*	223	83.41%
TESA	27	77.78%	*=1*	37	75.68%
**Hospital**	1,010	86.14%	<*0.01*	740	80.14%

Using a multiple nonlinear regression model, the influence of the following variables on the assessment of job satisfaction was tested as a dichotomous variable: gender, age, professional category, level of education, type of department and the unit of which the employee was a part. The type of department was considered a categorical variable and depending on where the employee carried out his activity was coded as follows: office - non-medical staff, surgical - ward with a surgical profile, clinic - ward with a non-surgical profile, laboratory - analysis laboratory; and radiology - radiology and imaging departments. The unit that the employee belonged to before the merger was also coded and entered into the model with two possible values: the employee belonged to the unit that was absorbed and the employee belonged to the other hospital unit. Using the *backward selection* method, all variables were introduced into the model and eliminated one by one according to the *p*-value returned by the model. The only variable influencing job satisfaction was the unit to which the staff belonged. This aspect is also observed in Table 6.

**Table 6:** The percentage of staff who are satisfied with their job satisfaction according to the unit they were part of before the merger.

**Sanitary unit**	**CCEHO**		**vs**	**MCHO**	
	**No**.	**% Satisfied**	***p***	**No**.	**% Satisfied**
Year 2022	551	90.02%	<*0.0001*	459	81.48%
Year 2023	366	80.60%	*=0.8*	374	79.68%

The employees belonging to the unit that was incorporated is associated with a lower percentage of people who declare themselves satisfied with job satisfaction, but the differences are statistically significant only for the year 2022, 90.02% vs 81.48% (χ^2^, *p* < 0.0001).

The structure of the questionnaire applied in 2023 allows the numerical assessment of job satisfaction. The scale used was from 1 to 5, with 1 being the value expressing total dissatisfaction and 5 being the value expressing total satisfaction. The values obtained for job satisfaction according to different variables are presented in Table 7.

**Table 7:** Job satisfaction one year and four months after the merger.

**Variable**	**Category**	**No**.	**Value**	**Applied test**
Professional category	Doctors	153	4.02	Anova test, *p* > 0.05
	Nurses	327	4.05	
	Aux. staff	223	4.14	
	TESA	37	4.08	
Sanitary unit	CCEHO	366	4.09	T test, *p* > 0.05
	MCHO	374	4.06	
Department type	Office	46	4.20	Anova test, *p* > 0.05
	Surgical ward	111	4.08	
	Medical ward	512	4.06	
	Laboratory	37	4.13	
	Radiology	34	4.05	
Sex	Women	618	4.09	T test, *p* > 0.05
	Men	122	4.00	
Education	Higher education	409	4.04	Anova test, *p* > 0.05
	Secondary education	306	4.11	
	General studies	25	4.15	
Age	<30	65	3.94	Anova test, *p* < 0.05
	31–40	135	4.03	
	41–50	259	4.05	
	51–60	170	4.20	
	>60	17	4.31	
Job satisfaction		740	4.07	

The job satisfaction of auxiliary staff is 4.14, this being the most satisfied personnel category in 2023. At the opposite pole are doctors who register the lowest value (4.08). However, the differences between staff categories are not statistically significant in terms of job satisfaction (Anova test, *p*>0.05). The health unit of which the employee belonged before the merger does not influence job satisfaction in 2023, with similar values for job satisfaction being recorded: employees who were part of the unit that incorporated = 4.09 and employees who were part of the unit integrated = 4.06 (*T*-test, *p*>0.05). Depending on the type of department the employee belongs to, there are slight differences in terms of job satisfaction. The most satisfied employees are those who work in an administrative department (office) with a value of 4.20. At the opposite pole is the employee from the radiology departments with a value of 4.05. Job satisfaction in medical (4.06) and surgical (4.08) departments is slightly lower than that of administrative staff, but the differences are not statistically significant (Anova test, *p*>0.05). Female staff (4.09) and staff with general education (4.15) show higher job satisfaction, but without statistical significance (Table 7). Job satisfaction is influenced by the age of the employee. Younger age is associated with lower job satisfaction. People under the age of 30 have a job satisfaction value of 3.94 compared to people over 60 who register a value of 4.31 (Tukey's HSD test, *p* < 0.05).

## 4 Discussion

The main aim of the study was to evaluate the job satisfaction of both medical and non-medical staff, after the merger of two public sector hospitals in Romania. We consider it necessary to mention a few particularities related to the context of the merger of the two hospitals. During the COVID-19 pandemic, MCHO was designated a covid hospital and CCEHO was a covid support hospital (March 2020–March 2022), the period preceding the merger process, by Order no. 533/2020 regarding the approval of the Plan of measures for the preparation of hospitals in the context of the COVID-19 coronavirus epidemic and the List of support hospitals for patients tested positive for the SARS-CoV-2 virus published in the Official Gazette, Part I no. 263 of March 31, 2020 Plan (2020). A previous study among CCEHO employees did not reveal negative effects of the pandemic on job satisfaction (Ilea et al., 2023). At the time of the merger (February 26, 2022), the structure of the two hospitals was joined, no wards were established or disbanded, and the number of beds in the new structure resulted from the summation of the number of beds in the two hospitals. In the first six months after the merger, wage increases related to working conditions were equalized; it should be mentioned that there were differences considering the fact that the legislation provides for the granting of increments between a minimum and a maximum, so that some employees registered a decrease in salary income. The salary increases initiated in the public health system in 2018 did not have the expected effects on the staff, not being correlated with an increase in the level of financial motivation for all categories of employees (OECD, 2019; Ilea et al., 2020).

Ten months after the merger, structural changes were made with the establishment or disbandment of wards/compartments and the reduction of approximately 100 beds, some of the staff being redistributed to other wards. Some of the medical staff and auxiliary staff was delegated to other wards to make up for the staffing requirements, due to the faulty rationalization and blocking of competitions for hospital posts. The responsibility of doctors to achieve the services contracted with the Health Insurance House (number of cases in continuous hospitalization, day hospitalization and ambulatory consultations) and to reduce the number of invalidated services, placed additional pressure on them. All these aspects contributed greatly to employee satisfaction.

The percentage of staff who rate job satisfaction at a high level is 86.14% ten months after the merger and 80.14% one year and four months after the merger. The results are in consistent with those presented in the specialized literature where most articles present a negative impact of mergers on job satisfaction (Lim, 2014). A study carried out in Italy, for both medical and administrative staff, one year after the merger, mentions a good level of job satisfaction in general, with more than 75% of employees considering that there is an improvement in the workplace (Isonne et al., 2021). The same study shows that administrative staff are more likely to have a lower job satisfaction score, opposite results to those found in this study (Isonne et al., 2021).

A statistically significant decrease is observed in the percentage of those who declare themselves contented with the general job satisfaction among doctors (Table 5). Opposite results were described in the study presented by Ka Keat Lim, which analyzes the effect of mergers produced in the UK health system during the period 2009–2011 on job satisfaction. The study describes an increase in job satisfaction in the first year after joining especially among physicians (Corrigan et al., 2012; Lim, 2014). Among nurses within the CCEHBh, a decrease in the percentage of those contented with job satisfaction is observed compared to 2022, from 86.03% to 81.96% in 2023, but it remains above the percentage observed among doctors (Table 5). Similar results are described in the literature, with nurses showing a decrease in job satisfaction following a merger process (Idel et al., 2003; Brown et al., 2006). Nurses represent a key category in major organizational changes because they represent the largest professional category that is present at the workplace practically without interruption (Jones, 2000). Nurses who received support from the hospital and the union had a higher level of job satisfaction (Burke and Greenglass, 2001).

The results obtained after the administration of the questionnaire are carefully analyzed by the hospital management. An important objective of the hospital is to achieve job satisfaction of the employed staff. Thus, after the presentation to the management of the analysis of the employee satisfaction questionnaire, by the designated work team, meetings/work sessions were held with the heads of sections/compartments/services. The purpose of these work meetings was to identify the causes of work dissatisfaction in order to find solutions to remedy the problems. The main causes that determined the dissatisfaction of employees were: the reward for the work performed (correlation of increments related to jobs), the way work is organized and carried out (definition of tasks, work performed) and the change in the style of supervision of the employees. This last cause was generated by a series of regulations/procedures implemented at the hospital level after the merger and reorganization. The solutions identified were: achieving better organizational communication and the informal circuit, revising the job description for all employees, reorganizing the workplace with the clear establishment of responsibilities per activity sector, continuous review and implementation of new work procedures and protocols, employees involvement in decision-making process. Also, the hospital management recommended the heads of departments/services/compartments to support the subordinate staff, considering the fact that the superior hierarchical head is in direct contact with the subordinates and has the responsibility for the proper performance of the work, increasing professional satisfaction and work results (Creangă and Gîtlan, 2013).

Effective communication, from the planning phase of a merger, with the employed personnel is essential (Ivancevich et al., 1987; Mocny, 2010). The percentage of CCEHBh employees who declare that there is a good collaboration with the management of the unit remains at a constant level. This fact is due to the proactive attitude of the Hospital's management, which makes every effort to streamline the communication process both directly (meetings, work meetings) and indirectly (internal memos, procedures). Improving the relationship between the unit's management and employees is essential for maintaining a high level of job satisfaction (Schön Persson et al., 2018; Isonne et al., 2021). Communication in the case of mergers is essential, the communication of the strategy, involvement and collaboration at all levels of the institution increasing the chances of success (Maile et al., 2022).

The aspect with the most dramatic drop in employee appreciation is job security. One year and 4 months after the merger, only a percentage of 53.38% declared themselves satisfied with this aspect. Similar results were reported by the study on the effects of mergers in the Italian healthcare system, which states that more than half of the participants are dissatisfied with the working conditions (Isonne et al., 2021). Another aspect that suffers a decrease is the career appreciation which is satisfactory in the year 2023 for 74.73% with a significant depreciation compared to the year 2022 (from 81.49%). Similar results are presented by the aforementioned study (Isonne et al., 2021). In the framework of mergers, the relocation of human resources is inevitable and implicitly some career paths will undergo changes, aspects that can lead to the appearance of dissatisfaction (Fulop et al., 2005; Isonne et al., 2021).

From a managerial perspective, occupational safety is a continuous concern of the hospital management. The investments of the last years aimed at rehabilitating and modernizing the premises, equipping them with medical equipment in order to improve the conditions, safety measures and protection at the workplace (Ilea et al., 2023). However, after the merger process between the two hospitals, employees value job security less. From the discussions held with the heads of departments/services/compartments, the causes of this phenomenon were identified as new investments and modern technologies which, although they make life easier and increase work productivity, produce additional stress for employees. Thus, regular staff training on new equipment and avoidance of adding additional responsibilities were recommended.

The main cause of the decrease in employee satisfaction with regard to career appreciation is the lack of performance-based pay for staff. In Romania, the legislation on the remuneration of staff paid from public funds (where the public hospital is also located) does not include differentiated remuneration based on performance criteria (LEGE, 2017). To limit this shortcoming, the hospital management performs monthly analyzes of the employees' activity, which are then presented in a joint meeting to the heads of departments/services/compartments, where the employees with the best results are nominated. The hospital also grants additional leave days for professional training and finances staff training courses.

The present study, by its structure, is observational and descriptive and lays the foundations for more in-depth analyzes regarding the management of major changes within public health facilities and beyond. The merger between the two health units, from the perspective of employee job satisfaction management, can be an example to follow because, as the results show, we can consider that it was successfully managed.

The measures taken contribute to the completion of knowledge in this field, especially for the health field in Eastern European countries where studies related to these aspects are rare.

Unfortunately, no data were found on the effects of the merger process in the healthcare sector in Romania on the job satisfaction of employees, this study being, to the best of the authors' knowledge, the first of its kind conducted in this country.

The second objective of the present study was to test a new satisfaction questionnaire that could be applied in Romanian hospitals. At the moment there is no standardized questionnaire that can be applied in the Romanian sanitary field. This questionnaire has proven its reliability, with factor analysis and internal reliability tests showing good results. It also meets the minimum requirements stipulated by National Agency for Quality Management in Health. The application of a standardized satisfaction questionnaire at the country level can highlight general problems of the medical staff by completing the picture of the health system with the perspective of the employees. Such analyzes carried out in many countries have highlighted, for example, the effects of the Covid-19 pandemic on job satisfaction (Berg, 2022; Wagner, 2023). The information obtained would be useful for solving general dissatisfactions with the medical system before they become large and distort the proper conduct of medical activities.

## 5 Conclusions

Job satisfaction one year and four months after the merger is 4.07 (out of a maximum of 5). A downward trend in the percentage of staff satisfied at work is observed, associated with the implementation of the restructuring and economic efficiency measures that took place after the merger process. This trend must be followed over time and requires a reaction from the hospital management. Younger age was associated with a lower level of job satisfaction. No significant difference was found in terms of job satisfaction, one year and four months after the merger, for staff from the merged unit (4.06) versus staff from the absorbing unit (4.09), which proves an effective process of standardization undertaken by the hospital management.

The questionnaire applied in 2023 is one that has proven validity and reliability, being a good starting point for creating a standardized questionnaire that could be implemented in the vast majority of hospitals in Romania. Also, the application of the questionnaire at an interval of 3–6 months would highlight the result of the implemented measures and the trend of employee satisfaction within CCEHBh.

## 6 Limitation

The analyzed questionnaire did not specifically track the merger phenomenon of the two hospitals. It did not include questions related to other aspects that are influenced by a merger process such as: stress, job insecurity, intention to leave the employer, etc. Another important limitation was not knowing the job satisfaction threshold for staff in the merged unit. Although the satisfaction questionnaires were distributed to the same employees, we cannot say that those who answered in the two moments in time are the same people.

## Data availability statement

The original contributions presented in the study are included in the article/supplementary material, further inquiries can be directed to the corresponding author.

## Author contributions

CI: Conceptualization, Methodology, Writing—original draft, Writing—review & editing. LD: Conceptualization, Methodology, Writing—original draft, Writing—review & editing. FM: Conceptualization, Methodology, Writing—original draft, Writing—review & editing. MD: Conceptualization, Methodology, Writing—original draft, Writing—review & editing. DT: Conceptualization, Methodology, Writing—original draft, Writing—review & editing. AP: Conceptualization, Methodology, Writing—original draft, Writing—review & editing.
